# SCOP/PHLPP1β in the basolateral amygdala regulates circadian expression of mouse anxiety-like behavior

**DOI:** 10.1038/srep33500

**Published:** 2016-09-19

**Authors:** Jun J. Nakano, Kimiko Shimizu, Shigeki Shimba, Yoshitaka Fukada

**Affiliations:** 1 Department of Biological Sciences, School of Science, The University of Tokyo, 7-3-1 Hongo, Bunkyo-ku, Tokyo 113-0033, Japan; 2 Department of Health Science, School of Pharmacy, Nihon University, 7-7-1, Narashinodai, Funabashi-shi, Chiba 274-8555, Japan

## Abstract

While disruption of the circadian clock triggers a spectrum of affective abnormalities, how the clock regulates mammalian emotionality remains unclear. Here, we characterized the time-of-day-dependent regulation of mouse anxiety-like behaviors. We show that anxiety-like behaviors are expressed in a circadian manner in mice and demonstrate that the clock machineries in the dorsal telencephalon (dTel) are required for the time-of-day-dependent regulation of anxiety-like behaviors. We identify suprachiasmatic nucleus circadian oscillatory protein (SCOP/PHLPP1β) as an essential intracellular signaling molecule mediating this temporal regulation downstream of the clock. Using viral-mediated, basolateral amygdala (BLA)-specific knockout of *Scop*, we demonstrate that deletion of SCOP in the BLA exerts anxiolytic effects on the elevated plus maze at early subjective night, thereby blunting the circadian variation in the anxiety-like behavior. We conclude that the circadian expression of SCOP in the BLA plays a key role in generating circadian rhythmicity in the anxiety-like behavior. Our results demonstrate SCOP as a regulator of anxiety-like behaviors and reveal its key roles in the anxiogenic functions of the BLA.

Approach and avoidance behavior constitutes a core component of animal decision-making, where, in mammals, emotionality shapes the animals’ behavioral tendencies to approach or avoid by modulating appetitive-aversive motivation^1^,^2^,^3^,^4^. Fear or anxiety, for example, elicits a set of defensive behavioral responses toward an imminent or anticipatory aversive stimulus, respectively^5^. Recent studies utilizing optogenetic tools have identified various important brain regions and circuitries underlying fear/anxiety responses^5^. The amygdala is thought to be central to the regulation of fear/anxiety, and activation of basolateral amygdala (BLA) projections to the centromedial amygdala (CeA) elicits anxiolytic responses, whereas somatic activation of BLA neurons is anxiogenic^6^. Nevertheless, how the regulatory machineries converge to effectuate emotional modulation of behavior remains largely unknown. Molecular mechanisms underlying anxiety regulation are much less well understood, and currently available anxiolytic medications produce their effects through pharmacological mechanisms of action yet to be fully understood^7^. In order to better understand mammalian anxiety, addressing its regulation from an alternative perspective is of importance.

In light of this, recent findings on the functional relationship between the circadian clock and emotionality-related behaviors could provide an important clue. The circadian clock is an organism’s internal pacemaker system with an intrinsic period of *circa* 24 hours, where the “master” clock in the hypothalamic suprachiasmatic nucleus (SCN) receives input from retinal photoreceptors and accordingly synchronizes peripheral clocks distributed throughout the body, driving diverse physiological phenomena. Dysfunctions of the circadian clock such as those arising from shift work or jet lag have been linked to a variety of mood disorders^8^. Conversely, abnormalities in the circadian rhythmicity of various physiological measures have been observed in patients diagnosed with major mood/anxiety disorders^9^,^10^. In rodents, perturbations of the circadian clock by means of surgical, genetic, pharmacological, light-induced, or behavioral manipulations lead to a spectrum of abnormalities in emotionality-related behaviors, including elevated or attenuated anxiety-like behaviors^11^.

Recent evidence points to a mechanism by which dysfunctions in the circadian clockwork lead to abnormal emotionality through aberrant dopaminergic activity in the ventral tegmental area (VTA), a major dopaminergic nucleus^12^,^13^. Despite the established roles of dopamine and other monoamine systems in anxiety regulation, their causality in mood/anxiety disorders and sufficiency in the regulation of emotionality *per se* have been questioned^14^,^15^,^16^,^17^. Furthermore, while these studies provide important insights into affective abnormalities arising from clock dysfunction, much remains unknown as to how the circadian clock maintains emotionality-related behaviors at physiological levels. In humans, both positive and negative affect are reported to display diurnal variation^18^, whereas excessive diurnal variations in mood states are a hallmark of major depressive and bipolar disorders^19^, implicating the physiological importance of precise time-of-day-dependent regulation of emotionality.

In the present study, we sought to unravel the mechanisms governing mammalian anxiety regulation and characterized temporal regulation of mouse anxiety-like behaviors by the circadian clock. We examined the involvement of SCOP (SCN circadian oscillatory protein), a signaling molecule originally identified as a gene product whose expression oscillates in a circadian manner in the rat SCN^20^. SCOP is a 183-kDa protein comprising pleckstrin homology (PH), leucine-rich repeat, protein-phosphatase 2C-like, glutamine-rich, and PDZ-binding domains, and SCOP has been shown to regulate a range of intracellular signaling pathways^21^,^22^,^23^. In the mouse hippocampus, SCOP plays an essential role in the consolidation of long-term object recognition memory^24^. Here, we describe SCOP-mediated time-of-day-dependent regulation of anxiety-like behaviors.

## Results

### Anxiety-like behaviors in wild-type mice are under circadian regulation

**Figure 1:**
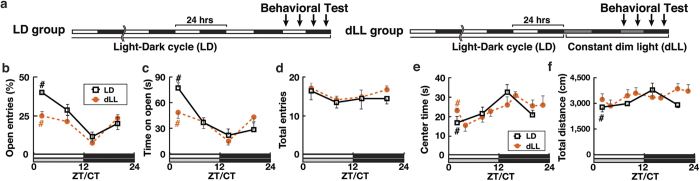
Diurnal and circadian expression of anxiety-like behaviors in wild-type mice on elevated plus maze (EPM, (**b**–**d**)) and open field (OF, (**e**,**f**)) tests. (**a**) Timeline for behavioral assays. LD: Mice were kept under an LD cycle and tested at one of 4 or 8 ZTs (Zeitgeber Time). dLL: Mice were placed under constant dim light 1 day prior to the testing and assayed at one of 4 or 8 CTs (Circadian Time). (**b–d**), Entries into open arms ((**b**), ratio of open:total entries), the time spent on open arms (**c**), and the total number of entries (**d**) for EPM. (**e**,**f**) The time spent in the center area (**e**) and the total distance traveled (**f**) for OF under LD (black) and dLL (orange) conditions. ^#^*P* < 0.05 between ZTs/CTs by one-way ANOVA. *n* = 6 per data point for EPM LD and OF LD, *n* = 12 per data point for EPM dLL, *n* = 7–12 for OF dLL.

In order to examine the temporal regulation of anxiety by the circadian clock, we profiled time-of-day-dependent variations in anxiety-like behaviors of wild-type (WT) mice. To evaluate mouse anxiety-like behaviors, we utilized the elevated plus maze (EPM) and open field (OF) tests. These paradigms are based on rodents’ intrinsic conflict between the drive to explore novel environments and the tendency to avoid open space; thus, increased time spent in the open arms of the EPM or in the center area of the OF is thought to represent reduced anxiety^25^,^26^. One group of mice (“LD” group, *n* = 24) were tested under a light-dark (LD) cycle at one of four or eight zeitgeber times (ZTs; lights on at ZT0, off at ZT12). The other group (“dLL” group, *n* = 76) were housed under a constant dim light condition (dLL) for >24 hrs and tested on the second day under dLL at one of four or eight projected circadian times (CTs; subjective day starts at CT0 and ends at CT12) (Fig. 1a). This enables us to eliminate the effects of cyclic light conditions. Testing CTs were projected using a circadian period (*τ*) of 25.0 hrs, as our mouse strains consistently exhibited free-running activity rhythms with a *τ* of 25.05 ± 0.07 hr SEM (*n* = 6). All mice were handled daily for >1 week prior to behavioral assays for acclimation. Housing under dLL had no observable effects on sleep/wake cycles or general activity rhythms.

In both the EPM and OF tests, anxiety-like behaviors exhibited diurnal variation and circadian rhythmicity under LD and dLL, respectively (Fig. 1b–f). Anxiety-like behaviors in the EPM and OF tests (hereafter referred to as “EPM-anxiety-like” and “OF-anxiety-like” behaviors, respectively) showed rhythms almost anti-phasic to each other: EPM-anxiety-like behavior was high at early subjective night (active phase) and low at early subjective day (resting phase), while OF-anxiety-like behavior was high at early subjective day and low at early subjective night (Fig. 1b,c,e). These results support the notion that EPM- and OF-anxiety-like behaviors reflect distinct physiological phenomena^25^,^26^,^27^ (See Discussion). No significant variation in the numbers of total arm entries or in general locomotor activity levels was found across the day except for OF total distance under LD (Fig. 1d,f), rendering it unlikely that circadian changes in general activity levels play a major role in the circadian expression of anxiety-like behaviors. The diurnal profiles were similar between the LD and dLL groups, suggesting that mouse anxiety-like behaviors are dynamically regulated by the intrinsic circadian clock rather than by external light conditions.

### The circadian clock in the dorsal telencephalon drives rhythmic anxiety-like behaviors

**Figure 2:**
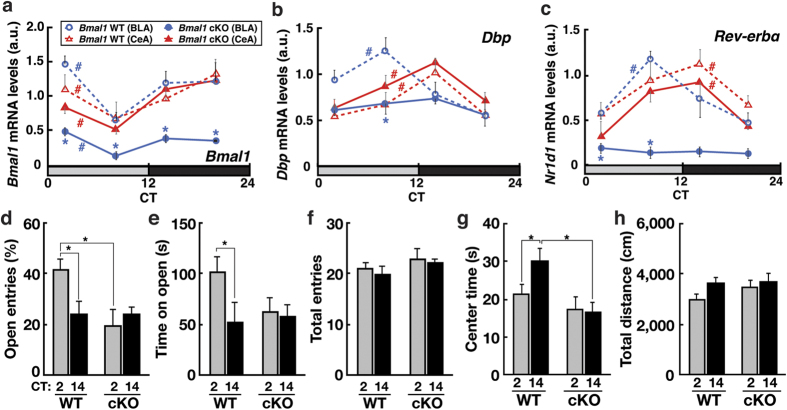
Circadian machineries in the dorsal telencephalon (dTel) regulate anxiety-like behaviors. (**a–c**) Circadian mRNA expression of clock genes *Bmal1* (**a**) *Dbp* (**c**) and *Rev-erb*α*/Nr1d1* (***C***) in the BLA (blue lines, circles) and CeA (red lines, triangles) of *Bmal1* cKO mice (“cKO”, *Bmal1*^*fl/fl*^ *Emx1*^*Cre/*+^) (solid lines) and littermate wild-type mice (“WT”, *Bmal1*^*fl/fl*^ *Emx1*^+*/*+^) (dashed lines) at CTs 2, 8, 14, 20. Data are normalized to *Rps29.* (**d**) % Open entries, (**e**) time spent on open arms, (**f**) Numbers of total arm entries in the EPM test for cKO and WT mice; (**g**) Center time and H, total distance traveled in the OF test for cKO and WT mice at CT2 (shaded bars) and CT14 (filled bars). (**a**–**c**) ^#^*P* < 0.05 among CTs by one-way ANOVA, *p < 0.05 vs WT at the corresponding CT. *n* = 3 per data point. (**d–h**) *p < 0.05 by unpaired Student’s *t*-test. *n* for WT: CT2, 9; CT14, 10. *n* for cKO: CT2, 8; CT14, 8. Data are means with SEM.

To examine the regulation of anxiety-like behaviors by the circadian clock, we used *Bmal1*^*fl/fl*^ *Emx1*^*Cre/*+^ conditional knockout (cKO) mice. *Emx1*^*Cre*^ expression is restricted to glutamatergic neurons and astrocytes in the dorsal telencephalon (dTel), which includes the neocortex, hippocampus, and BLA^28^. *Bmal1*^*fl/fl*^ *Emx1*^*Cre/*+^ mice lack BMAL1 protein in the dTel^29^. BMAL1 and CLOCK, both bHLH transcription factors, heterodimerize to activate E-box-mediated transcription and hence constitute the core transcriptional-translational feedback loop of the molecular circadian clock. Cells lacking BMAL1 lose circadian rhythmicity^30^. This cKO enables us to eliminate the effects from clock dysfunctions in the SCN, which leads to a systemic loss of circadian rhythmicity, and in the monoamine-producing nuclei in the midbrain. We first examined the effects of *Bmal1* cKO on the circadian expression of clock genes in amygdala subnuclei: BLA, a dTel subnucleus involved in the regulation of anxiety-like behaviors^5^,^6^, and CeA, a ventral telencephalic subnucleus. Both in the BLA and CeA of littermate WT mice, mRNA levels of *Bmal1*, *Dbp*, and *Rev-erb*α exhibited circadian variations (Fig. 2a–c). In the BLA of *Bmal1* cKO mice, *Bmal1* mRNA levels were downregulated by >3 fold, and *Dbp* and *Rev-erb*α expression was constantly low across the day (Fig. 2a–c, blue). Clock gene expression in the CeA was unaffected by *Bmal1* cKO (Fig. 2a–c, red), consistent with the lack of *Emx1* expression in the CeA^28^.

*Bmal1* cKO mice exhibited normal activity rhythms (Supplementary Fig. 1) and appeared physically normal. Whereas littermate WT mice (*Bmal1*^*fl/fl*^ *Emx1*^+*/*+^) reproduced the circadian variations in anxiety-like behaviors of WT mice (Fig. 1) at CT2 (early subjective day) and CT14 (early subjective night), anxiety-like behaviors remained high in *Bmal1* cKO mice both during the day and night at levels comparable to the peak levels in littermate WT mice (Fig. 2d–h). Circadian variations in anxiety-like behaviors between CT2 and CT14 were not observed in *Bmal1* cKO mice (Fig. 2d,e,g). *Bmal1* cKO had no significant effect on general locomotor activities (Fig. 2f,h).

### SCOP is expressed in a circadian manner in the amygdala

**Figure 3:**
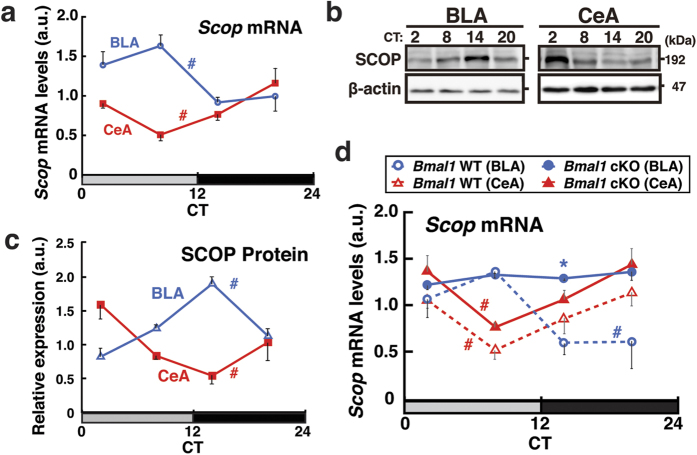
SCOP is rhythmically expressed in the amygdala. (**a–c**) Levels of *Scop* mRNA ((**a**) quantitative PCR) and SCOP protein ((**b**,**c**) immunoblotting) in the BLA (blue) and CeA (red) at CTs 2, 8, 14, 20. Representative immunoblots against SCOP and β-actin (**b**) and quantification of all samples (**c**) are shown. (**d**) *Scop* mRNA levels in the BLA (blue lines, circles) and CeA (red lines, triangles) of *Bmal1* cKO (solid lines) and littermate WT (dashed lines) mice, normalized to *Rps29.*^#^*P* < 0.05 between CTs by one-way ANOVA. *n* = 3 per data point. Data are means with SEM.

Given the involvement of the circadian clock in the temporal regulation of anxiety-like behaviors, we examined the roles of SCOP, a clock-controlled signaling protein. We first examined the expression of SCOP protein in dTel subregions. The prefrontal cortex, hippocampus, and BLA appeared to express SCOP in a circadian manner with higher expression at night (Supplementary Fig. 2). We then narrowed our focus on the BLA, a region known to play major roles in anxiety regulation^5^,^6^, and more fully profiled SCOP expression. Both mRNA and protein levels of SCOP exhibited circadian variations in the BLA, with mRNA peaking at CT8 and protein peaking at CT14 (Fig. 3a–c, blue). SCOP was also rhythmically expressed in the CeA, exhibiting a profile anti-phasic to that in the BLA (Fig. 3a–c, red), consistent with an earlier study reporting anti-phasic diurnal expression of the clock component PERIOD2 between the BLA and CeA^31^. In the BLA of *Bmal1* cKO mice, *Scop* mRNA levels were constantly high across the day with no significant circadian rhythmicity (Fig. 3d). Combined with the constantly low expression of E-box-regulated clock genes *Dbp* and *Rev-erb*α across the day in the BLA of *Bmal1* cKO mice (Fig. 2g,h), these results suggest indirect *Bmal1-*mediated transcriptional repression of *Scop* presumably through REV-ERB-mediated repression at the REV-ERB binding sites found in the intron 1 of *Scop* gene^32^, a genomic region conserved among placental mammals.

### SCOP in the dorsal telencephalon is required for circadian expression of anxiety-like behaviors

**Figure 4:**
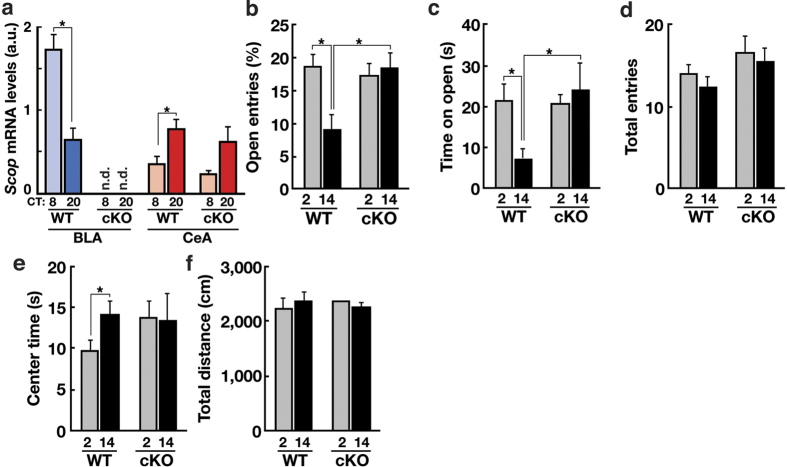
SCOP in the dTel is required for circadian expression anxiety-like behaviors at CTs 2 and 14. (**a**) *Scop* mRNA levels in the BLA (blue) and CeA (red) of cKO and WT mice at CT8 (light color) and CT20 (dark color). n.d., not detected. (**b**) % Open entries (**c**) time spent on open arms (**d**) Numbers of total arm entries for *Scop* cKO mice (“cKO”, *Scop*^*fl/fl*^ *Emx1*^*Cre/*+^) and littermate WT mice (“WT”, *Scop*^*fl/fl*^ *Emx1*^+*/*+^). (**e**) Center time and (**f**) total distance traveled for cKO and WT mice. *p < 0.05 by unpaired Student’s *t*-test n.d.: not detected. (**a–e**) *n* for WT: CT2, 14; CT14, 13. *n* for cKO: CT2, 9; CT14, 9. (**f**) *n* = 3 per data point. Data are means with SEM.

To examine the involvement of SCOP in anxiety regulation, we used dTel-specific *Scop* cKO mice (*Scop*^*fl/fl*^ *Emx1*^*Cre/*+^)^29^. In the BLA, *Scop* mRNA levels were undetectable both at CT8 and CT20 in *Scop* cKO mice (Fig. 4a, blue), whereas *Scop* expression in the CeA was not significantly affected (Fig. 4a, red). *Scop* cKO mice exhibited no observable defects in circadian activity rhythms or sleep/wake cycles^29^ or in general locomotor activity (Fig. 4d,f). Circadian expression of *Bmal1* was intact in the BLA and CeA of *Scop* cKO mice (Supplementary Fig. 3). In both the EPM and OF tests, *Scop* cKO mice failed to express circadian changes in anxiety-like behaviors: While littermate WT mice (*Scop*^*fl/fl*^ *Emx1*^+*/*+^) reproduced the circadian variations in anxiety-like behaviors of WT mice (Fig. 1) between CT2 (day) and CT14 (night), anxiety-like behaviors remained constantly low at both CTs in *Scop* cKO mice (Fig. 4b–f), a phenotype distinct from that of *Bmal1* cKO mice (Fig. 2). This is in line with the elevated *Scop* expression observed in the BLA of *Bmal1* cKO mice (Fig. 3d), leading us to hypothesize that changes in SCOP levels in the BLA have direct effects on anxiety-like behaviors.

### SCOP in the BLA has an anxiogenic function and is essential for circadian expression of anxiety-like behavior in the EPM test

**Figure 5:**
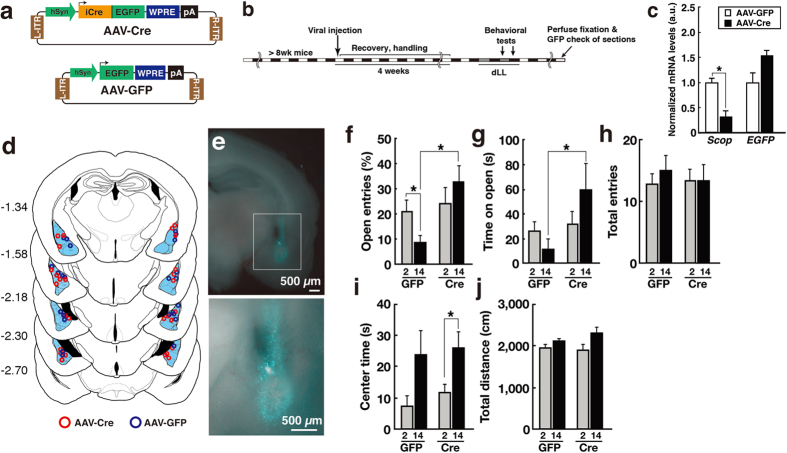
SCOP in the BLA is required for the circadian changes in EPM-anxiety-like behavior. (**a**) AAV vectors expressing iCre::EGFP fusion protein (“AAV-Cre”) or EGFP (“AAV-GFP”) were constructed (See smentary Information). hSyn: human synapsin promoter; WPRE: woodchuck hepatitis virus posttranscriptional regulatory element; pA: poly-A; ITR: inverted terminal repeat. (**b**) Timeline for the viral injection, behavioral tests (EPM and OF) and histological inspection. (**c**) *Scop* and *EGFP* mRNA levels in the BLA of *Scop*^*fl/fl*^ mice injected with AAV-GFP (open bars) or AAV-Cre (filled bars). (**d**) Histological reconstruction of the injection sites in coronal sections of the mouse brain. Values are distances in millimeters from Bregma (minus indicates a position posterior to Bregma) according to the mouse stereotaxic atlas by Paxinos and Watson^47^. AAV-Cre (magenta) or AAV-GFP (blue) was injected bilaterally to the BLA (shown in cyan) for each hemisphere of an individual mouse. Each colored circle represents the site of the strongest GFP signal. (**e**) A representative image of the infection are in the BLA. (**f–j**) Anxiety-like behavior in BLA-specific *Scop* KO mice. EPM % Open entries (**f**), time spent on open arms (**g**), total numbers of entries into closed and open arms (**h**), OF center time (**i**), and OF Total distance (**j**) at CT2 (shaded bars) and CT14 (filled bars) for mice bilaterally infected with AAV-Cre (“Cre”) and mice bilaterally infected with AAV-GFP (“GFP”). *p < 0.05 by unpaired Student’s *t*-test. *n* for behavioral assays: Cre, 6 (CT2) and 8 (CT14) for EPM, 8 (CT2) and 6 (CT14) for OF; GFP, 6 (CT2) and 6 (CT14). Data are means with SEM. Scale bars, 500 μm.

To investigate the function of SCOP in the BLA, we examined the effects of *Scop* knockout (KO) in the BLA. To this end, we constructed adeno-associated virus (AAV) expressing Cre recombinase fused to EGFP (“AAV-Cre”) or EGFP alone (“AAV-GFP”) driven under human synapsin (hSyn) promoter (Fig. 5a). *In vitro* analyses using cultured cells confirmed the recombinase activity of the fusion protein, Cre expression in neuronal cells, and the infectious ability of the viral constructs in neurons (Supplementary Fig. 4). We then injected AAV-Cre or AAV-GFP bilaterally into the BLA of *Scop*^*fl/fl*^ mice (*Scop* BLA KO) and examined the effects of BLA-specific *Scop* KO on anxiety-like behaviors (Fig. 5b). Transduction with AAV-Cre resulted in a >3 fold reduction in *Scop* mRNA levels in the BLA of *Scop*^*fl/fl*^ mice compared to the BLA of *Scop*^*fl/fl*^ mice transduced with AAV-GFP (Fig. 5c–e). Behaviorally, -BLA-specific *Scop* KO mice phenocopied *Scop* cKO mice in the EPM test, exhibiting lower anxiety-like behavior both at CT2 and CT14 (Fig. 5f–h). BLA-specific *Scop* KO abrogated the circadian variation in open arm entries in the EPM test between the CTs (Fig. 5f,g). The effects of BLA-specific *Scop* KO on open arm entries at CT14 were significant compared to mice bilaterally transduced with AAV-GFP. The total numbers of entries were unaffected (Fig. 5h). AAV-GFP-transduced mice exhibited circadian changes in open arm entries at levels comparable to that in wild-type (Fig. 1b) or *Scop*^*fl/fl*^ mice (Fig. 4b). BLA-specific *Scop* KO had no statistically significant effect on anxiety-like behavior in the OF test (Fig. 5i,j), suggesting region-specific roles of SCOP in regulating anxiety-like behaviors in the EPM and OF tests (See Discussion). These results demonstrate that SCOP in the BLA functions to elevate anxiety-like behavior in the EPM test at early subjective night (CT14), when SCOP level in the BLA peaks.

## Discussion

Our results demonstrate that the anxiogenic function of SCOP in the BLA drives circadian rhythms in EPM-anxiety-like behavior in mice. We show that the clock machinery and SCOP in the dTel are required for the circadian expression of anxiety-like behaviors in both the EPM and OF tests (Figs 2 and 4). *Bmal1* cKO leads to upregulation of SCOP expression in the BLA (Fig. 3d) and has anxiogenic effects, whereas *Scop* cKO has anxiolytic effects. Deletion of SCOP in the BLA abolishes the diurnal elevation of EPM-anxiety-like behavior at early subjective night (CT14) (Fig. 5f,g), signifying the role of SCOP in the BLA in driving the anxiety-like behavior in the EPM test. Collectively, we conclude that SCOP is a regulator of mouse anxiety-like behaviors functioning in anxiogenesis, driving circadian changes in these behaviors, and that the rhythmic expression of SCOP in the BLA is required for the circadian expression of anxiety-like behavior in the EPM test.

BLA-specific deletion of SCOP resulted in a loss of the circadian variation in anxiety-like behaviors in the EPM test (Fig. 5f,g) but not in the OF test (Fig. 5i,j), while dTel-specific deletion of SCOP abolished the rhythms in both behaviors (Fig. 4b–f). These data suggest that while SCOP in the dTel is required for the circadian variation in anxiety-like behaviors in the OF test, SCOP in the BLA, which is part of the dTel, is not. It is therefore likely that a dTel subregion(s) other than the BLA is responsible for the circadian regulation of anxiety-like behaviors in the OF test.

Our results also demonstrate an anti-phasic relationship in the circadian expression of SCOP in the BLA and CeA (Fig. 3). Antiphasic expression of the clock gene *Period2* in these amygdala nuclei have previously been reported^31^, and our data demonstrate that these nuclei also exhibit distinct phase distributions in the circadian expression of clock genes *Bmal1*, *Rev-erbα*, and *Dbp* (Fig. 2a–c). Previous studies have reported roles of tissue-specific transcriptional co-regulators, such as CBP/p300, in regulating tissue- and cell type-specific circadian transcription and phase distributions^33^,^34^. We thus speculate that BLA- and CeA-specific co-activators and/or co-repressors may regulate distinct rhythms in mRNA and protein levels of SCOP and clock genes, although we have been unable to directly examine protein levels of core clock genes in the amygdala subregions potentially due to their low abundance.

SCOP is a clock-controlled multi-domain signaling molecule regulating various intracellular signaling pathways^20^,^21^,^22^,^23^,^24^,^35^. Notably, SCOP and its shorter variant PHLPP1α have been shown to dephosphorylate AKT and thereby suppress PI3K/AKT/GSK3β signaling, where signaling cascades downstream of dopamine D_2_, 5-HT_1A_ and 5-HT_2_ receptors converge^36^. In humans and mice, AKT1, AKT2, and GSK3β have been associated with abnormal anxiety^37^,^38^,^39^. These data point to a model in which SCOP regulates anxiety by modulating AKT/GSK3β signaling. In addition, SCOP has been shown to regulate K-Ras/ERK signaling in the hippocampus^24^,^40^. MAPK/ERK signaling in the amygdala has been repeatedly shown to contribute to anxiety regulation in rodents. For example, exposure to novel environments or repeated intraperitoneal injection of corticosterone, which enhances anxiety-like behavior in the EPM test, increases ERK activation in the rat amygdala^41^,^42^, whereas ERK inhibition in the mouse BLA abolishes restraint stress-induced increase in anxiety-like behavior in the EPM test^43^. Given the crosstalk reported for MAPK/ERK and AKT/GSK3β signaling pathways, it is therefore of interest to examine how SCOP regulates these anxiety-related signaling pathways in the BLA *in vivo*.

Importantly, the anxiogenic and anxiolytic effects of *Bmal1* and *Scop* cKO, respectively, did not exceed the range of the circadian variations of anxiety-like behaviors expressed by wild-type mice (Figs 2 and 4). Put another way, the loss of circadian rhythmicity in the dTel appears to “halt” the circadian expression of anxiety-like behaviors at a specific time of day. We therefore speculate that the circadian clock in the dTel confers circadian rhythmicity on mouse anxiety-like behaviors. This, together with our finding that the anxiety-like behaviors in the EPM and OF tests peak at distinct times of day (Fig. 1b,c,e), suggests that the rhythmic expression of anxiety-like behaviors may play an important survival function. In this regard, examining whether and how this circadian expression is conserved in other species, especially in diurnal animals, will be of particular interest.

It has to be noted that the distinct temporal profiles in the expression of anxiety-like behaviors in the EPM and OF tests are not an unexpected finding, as these behaviors have been frequently suggested to reflect distinct physiological phenomena. For example, factor analysis studies using rodents revealed that anxiety-related behaviors evaluated in the EPM and OF tests do not load on a common factor^44^,^45^. Consistently, multiple pharmacological manipulations have been shown to produce test-selective effects on these anxiety-like behaviors^46^. Hence, a current model considers the anxiety-like behaviors evaluated in the EPM and OF tests, as well as in other anxiety-related tests, as representing partially overlapping but distinct aspects of the multidimensional “anxiety”, likely underlain by partially overlapping mechanisms^26^. Not only are our results (Fig. 1b,c,e) compatible with such a model, our finding that clock dysfunction in the dTel abolishes the distinct phase relationship between the EPM- and OF-anxiety-like behaviors (Fig. 2) suggests that the circadian clock may be actively involved in the differentiation of these anxiety-like behaviors. Although further study is necessary to elucidate the underlying mechanisms, these results, combined with the selective effects of *Scop* BLA KO on the EPM but not the OF test (Fig. 5f–j), could provide a foundation for unraveling the complexity of anxiety-like behaviors.

In summary, we have described the circadian expression of mouse anxiety-like behaviors, which requires clock machineries and SCOP function in the dTel, and demonstrated that SCOP in the BLA is expressed in a circadian manner and exerts anxiogenic effects on the EPM. While the molecular mechanisms remain to be established, we conclude that circadian regulation of SCOP levels in the dTel plays an essential role in generating the circadian rhythmicity in anxiety-like behaviors, which could represent an important function in animal survival. By characterizing the involvement of a temporal axis in the regulation of mammalian anxiety-like behavior, as well as by identifying key molecular and neuroanatomical players therein, our present study provides a new foundation for future research on animal emotionality. Further understanding of the intrinsic plasticity in anxiety regulation described here could potentially enable the development of new classes of treatment for anxiety/mood disorders.

## Methods

### Animals and housing

All experiments were conducted in accordance with guidelines set by The University of Tokyo and approved by the Committee on Animal Care and Use of the Graduate School of Science at The University of Tokyo. Wild-type male C57BL/6J mice were purchased from Tokyo Laboratory Animals Science Co., Ltd (Tokyo, Japan). *Emx1*^*Cre/*+* *^28 mice were kindly provided by Dr. Atsu Aiba. All animals were initially housed under a 12 hr light/12 hr dark cycle (lights on at 8 am) in temperature- and humidity-controlled compartments with food and water available *ad libitum*. More information is available in Supplementary Information.

### Surgery

Male *Scop*^*fl/fl*^ mice aged 8–10 weeks were deeply anesthetized with a mixture of ketamine (140 mg/kg) and xylazine (8.8 mg/kg) in bacteriostatic saline given intraperitoneally (20 ml/kg) and placed on a stereotactic apparatus (Narishige, Tokyo, Japan). The skull was exposed, and holes were drilled bilaterally above the basolateral amygdala. The coordinates relative to bregma were: anteroposterior, −1.65 mm; lateral, +3.30 mm; dorsoventral, −4.45 mm. Mice were bilaterally injected with 0.5 μl of either AAV-Cre or AAV-GFP over 5 min, and the needles were kept in place for an additional 5 min to ensure infusion.

### Behavioral assays

Male mice aged 12–16 weeks (wild-type and AAV-injected *Scop*^*fl/fl*^) or 20–25 weeks (*Bmal1* and *Scop* cKO) were subjected to behavioral tests. Littermate *Bmal1*^*fl/fl*^ *Emx1*^+*/*+^^46^ or *Scop*^*fl/fl*^ *Emx1*^+*/*+^ mice were used for control. Prior to testing, all mice were singly housed, entrained to the LD cycle for >2 weeks, and handled daily for acclimation at random times of day for >1 week. All behavioral assays were conducted under dim light at 4.0 ± 0.1 lux at the center of the apparatuses. Mice were picked up on the operator’s palms and released into the apparatuses such that the mice voluntarily walk into the maze or open field from the palms. For BLA-specific KO experiments, behavioral data from mice with clear bilateral GFP signal in the basolateral amygdalar complex were adopted as AAV-Cre or AAV-GFP data. For fluorescent microscopy, AAV-injected mice were sacrificed by rapid cervical dislocation, and the brain was removed, mounted on a brain matrix (ASI Instruments), and sliced into 1-mm thick sections. The sections were analyzed for GFP signal under a fluorescent stereoscopic microscope (Leica). Detailed procedures are described in Supplementary Information.

### Elevated Plus Maze

Mice were placed in the center of an elevated plus maze (O’Hara & Co., Ltd., Tokyo, Japan) facing one of the closed arms. The maze has four arms (5 × 25 cm), the opposing two of which are protected with clear walls (16 cm high), and is elevated 50 cm from the ground. Mice were allowed to freely explore the maze for 5 min; their behavior was monitored with an automated video tracking system, and the time spent on open arms and entries into open/closed arms were determined using TIME_EP software (O’Hara & Co., Ltd.) or manually by an analyst blinded to time-of-day information (see Data analysis).

### Open Field

Mice were placed into one of the corners of a 45 × 45 cm open field (O’Hara & Co., Ltd.), and their behavior was monitored for 5 min with an automated video tracking system. The time spent in the center of the field (30% area, circular) and the distance traveled were determined using ImageJ software (NIH, MD) with OpenField plug-in (O’Hara & Co., Ltd.).

### Data analysis

Behavioral analyses were automated except for the elevated plus maze (EPM) test for wild-type C57BL/6J mice, for which all recorded behavioral data were shuffled into random orders, and entries into open and closed arms and the time spent on each arm were recorded by an operator blinded to time-of-day information for each subject. One-way ANOVA tests were used to examine the statistical significance of data consisting of three or more groups. Unpaired two-tail Student’s *t*-tests were used for analysis on data with two groups. All data are presented as means with SEM. *P* values are presented as “*P*” for ANOVA tests and “*p*” for *t*-tests.

## Additional Information

**How to cite this article**: Nakano, J. J. *et al*. SCOP/PHLPP1β in the basolateral amygdala regulates circadian expression of mouse anxiety-like behavior. *Sci. Rep.* **6**, 33500; doi: 10.1038/srep33500 (2016).

## Supplementary Material

Supplementary Information
