# Supplementary material for: SCOP/PHLPP1β in the basolateral amygdala regulates circadian expression of mouse anxiety-like behavior
Source: Sci Rep. 2016 Sep 19;6:33500. doi: 10.1038/srep33500 (PMC5027591; doi:10.1038/srep33500)
Supplement: Supplementary Information [file srep33500-s1.pdf]

Supplementary Information for

SCOP/PHLPP1β in the basolateral amygdala regulates circadian expression of mouse anxiety-like behavior

Jun J. Nakano[1], Kimiko Shimizu[1,*], Shigeki Shimba[2], Yoshitaka Fukada[1,*]

[1]Department of Biological Sciences, School of Science, The University of Tokyo, 7-3-1 Hongo, Bunkyo-ku, Tokyo 113-0033, Japan

[2]Department of Health Science, School of Pharmacy, Nihon University, 7-7-1, Narashinodai, Funabashi-shi, Chiba 274-8555, Japan

[*]Correspondence should be addressed to:
shimizuk@bs.s.u-tokyo.ac.jp (K.S.)
sfukada@mail.ecc.u-tokyo.ac.jp (Y.F.)

Supplementary Methods:

Plasmids. pCAG-iCre was a generous gift from Dr. Takahiko Matsuda (Kyoto University Institute for Virus Research, Kyoto, Japan). The pCCALL2 vector was a generous gift from Dr. Andras Nagy (Samuel Lunenfeld Research Institute, Mount Sinai Hospital, Toronto, Canada). pAAV-hSyn-hChR2(H134R)-EYFP was a gift from Karl Deisseroth (Addgene #26973). To produce pCCALL2-mCherry, a fragment containing the *mCherry* gene from *ins:mCherry*[1] was inserted into pCCALL2 linearized with *Xho*I and *Bgl*II.

Animals and housing. All experiments were conducted in accordance with guidelines set by The University of Tokyo and approved by the Committee on Animal Care and Use of the Graduate School of Science at The University of Tokyo. Wild-type male C57BL/6J mice were purchased from Tokyo Laboratory Animals Science Co., Ltd (Tokyo, Japan). To obtain dorsal telencephalon (dTel)-specific *Bmal1*$^{fl/fl}$ *Emx1*$^{Cre/+}$ conditional knockout (cKO) mice (Fig. S1), *Bmal1*$^{fl/fl}$ mice[2] were crossed with *Emx1*$^{Cre/+}$ mice[3,4], kindly provided by Dr. Atsu Aiba (Center for Disease Biology and Integrative Medicine, The University of Tokyo, Japan). To obtain *Scop*$^{fl/fl}$ *Emx1*$^{Cre/+}$ conditional knockout (cKO) mice *Scop*$^{fl/fl}$ mice (K. Shimizu, *et al.*, *Nat Commun* 2016, in press) were crossed with *Emx1*$^{Cre/+}$ mice. Briefly, *Scop*$^{fl/fl}$ mice were generated by flanking the exon 4 of *Scop* (*Phlpp1*) gene with *loxP* sequences; Cre-mediated recombination results in a frameshift mutation and a premature stop codon, resulting in SCOP deletion (K. Shimizu *et al.*, *Nat Commun* 2016, in press). *Bmal1*$^{fl/fl}$ mouse line was on a C57BL/6J background; *Scop*$^{fl/fl}$ mouse line was on a C57BL/6N background. All animals were initially housed under a 12 hr light/ 12 hr dark

cycle (lights on at 8 am) in temperature- and humidity-controlled compartments with food and water available *ad libitum*.

Adeno-associated virus (AAV) vector production. To produce iCre::EGFP fusion protein construct, the *EGFP* gene was PCR amplified from pEGFP-C1 (Addgene 2487) using the primer set: 5'-CTGCT CGAGG ATGGG GACGG ACCGG TCGCC ACCAT GGTGA GCAAG-3' and 5'-CGAGG CGGCC GCTAT TACTT GTACA GCTCG TCCAT GCCGA GAGTG-3'. The fragment was digested with *Xho*I and *Not*I and inserted into pCAG-iCre digested with *Xho*I and *Not*I to produce pCAG-iCre::EGFP. The *iCre::EGFP* fragment including the nuclear-localization signal (NLS) was then PCR amplified using the primer set: 5'-CTGGA ATTCG CGGCC GCTAT TACTT GTACA GCTCG TC-3' and 5'-TTCGG ATCCG CCACC ATGGT GCCCA AGAAG AA-3'. The fragment was then digested with *Bam*HI and *Eco*RI and inserted into the 4.5-kb fragment of pAAV-hSyn-hChR2(H134R)-EYFP digested with *Bam*HI and *Eco*RI to obtain pAAV-hSyn-iCre::EGFP-WPRE ("pAAV-Cre"). The resulting vector encodes a fusion protein of iCre and EGFP with a 5 amino acid linker in between (GPVAT); EGFP fusion at the C-terminus has been shown to not interfere with Cre recombinase activity[5] (See also Fig. S4*A,B*). To produce pAAV-hSyn-EGFP-WPRE ("pAAV-GFP"), the *EGFP* gene was PCR amplified from pEGFP-C1 using the primer set: 5'-CGCTG GATCC ACCGG TCGCC ACCAT GGTGA GCAAG-3' and 5'-AGATG AATTC TATTA CTTGT ACAGC TCGTC CATGC CGAGA GTG-3'. The fragment was then digested with *Bam*HI and *Eco*RI and inserted into the 4.5-kb fragment of pAAV-hSyn-hChR2 (H134R)-EYFP digested with *Bam*HI and *Eco*RI.

To validate the functionality of iCre::EGFP fusion protein, pCAG-iCre::EGFP was transfected to HEK293T/17 cells with pCCALL2-mCherry, which contains floxed β*-geo* (*lacZ*/neomycin-resistance fusion gene) cassette in front of *mCherry* gene.

Upon Cre-mediated recombination, the *β-geo* gene is excised out, and mCherry is expressed. pCAG-iCre served as a control for recombinase activity. Transfection was carried out using a standard polyethylenimine (PEI) method. Briefly, DNA (500 µg per 100-mm culture dish) was diluted in dH_2O, mixed with PEI (100 µg/mL final), incubated for 15 min at RT, and added to the culture medium. GFP and mCherry intensities were examined 48 hours after transfection under an inverted fluorescent microscope (Keyence, Osaka, Japan). To verify that the human synapsin (hSyn) promoter induces Cre expression in neuronal cells, Neuro-2a (N2a) cells were transfected with pAAV-Cre or pAAV-GFP using Lipofectamine 3000 reagent according to the manufacturer's instructions (Invitrogen, Carlsbad, CA, USA). GFP and mCherry intensities were examined as described above.

Validated pAAV vectors were then transfected to HEK293T/17 cells, together with pACG2-Y730F[6] and pHelper (Stratagene) plasmids, using a standard PEI method. Cells were harvested 72 hrs after transfection, collected in PBS, pelleted, and resuspended in 200 µl of Dulbecco's PBS per 100-mm culture dish (60 cm^2). After 3 freeze-thaw cycles in liquid nitrogen and 37°C water bath followed by a centrifugation, the lysate was treated with 1.25 U/µL Benzonase (Merck, Darmstadt, Germany) for 30 min at 37°C and then subjected to serial centrifugation until the supernatant was clear. The final purified viruses were aliquoted and stored at -80°C. The titers of our purified viral vectors were as follows: AAV-Cre, 8×10^{12}; AAV-GFP, 1×10^{13} genome copies/mL.

To verify the infectious ability, purified AAV viral vectors were transduced to primary hippocampal neurons. Primary hippocampal cultures were prepared as previously described[7]. Purified AAV-Cre or AAV-GFP was added to primary cultures at 20 days *in vitro* (DIV), and GFP signal was examined at 40 DIV as described above.

Surgery. Male *Scop*$^{fl/fl}$ mice aged 8-10 weeks were deeply anesthetized with a mixture of ketamine (140 mg/kg) and xylazine (8.8 mg/kg) in bacteriostatic saline given intraperitoneally (20 ml/kg) and placed on a stereotactic apparatus (Narishige, Tokyo, Japan). The skull was exposed, and holes were drilled bilaterally above the basolateral amygdala. The coordinates relative to bregma were: anteroposterior, -1.65 mm; lateral, +3.30 mm; dorsoventral, -4.45 mm. Mice were bilaterally injected with 0.5 µl of either AAV-iCre::EGFP or AAV-EGFP over 5 min, and the needles were kept in place for an additional 5 min to ensure infusion.

Behavioral assays. Male mice aged 12-16 weeks (wild-type and AAV-injected *Scop*$^{fl/fl}$) or 20-25 weeks (*Bmal1* and *Scop* cKO) were subjected to behavioral tests. Littermate *Bmal1*$^{fl/fl}$ *Emx1*$^{+/+}$ or *Scop*$^{fl/fl}$ *Emx1*$^{+/+}$ mice were used for control. Prior to testing, all mice were singly housed, entrained to the LD cycle for >2 weeks, and handled daily for acclimation at random times of day for >1 week. Mice that underwent stereotactic surgeries were allowed to recover for 4 weeks before the behavioral tests. All behavioral assays were conducted under dim light at 4.0 ± 0.1 lux at the center of the apparatuses. Mice were picked up on the operator's palms and released into the apparatuses such that the mice voluntarily walk into the maze or open field from the palms. Each mouse received one trial on each behavioral paradigm, with the elevated plus maze test always preceding the open field test. Mice were re-entrained to a 12 hr light/ 12 hr dark cycle (lights on at 10 am) for at 2 days following the elevated plus maze test under constant dim light before they were subjected to the open field test. The order of mice and their testing CT were randomized between assays (e.g. mice assayed in the open field test at CT2 consist of ones that have been assayed in the plus maze test at any of the 4 CTs). Apparatuses were cleaned and dried after every experimental trial. For BLA-specific KO experiments, behavioral data of AAV-injected

mice were sorted according to their infection sites determined by fluorescent microscopy (see below). Behavioral data from mice with clear bilateral GFP signal in the basolateral amygdalar complex (BLA; comprising lateral, basolateral, basomedial nuclei) were adopted as AAV-Cre or AAV-GFP data.

Elevated Plus Maze. Mice were placed in the center of an elevated plus maze (O'Hara & Co., Ltd., Tokyo, Japan) facing one of the closed arms. The maze has four arms (5 x 25 cm), the opposing two of which are protected with clear walls (16 cm high), and is elevated 50 cm from the ground. Mice were allowed to freely explore the maze for 5 min; their behavior was monitored with an automated video tracking system, and the time spent on open arms and entries into open/closed arms were determined using TIME_EP software (O'Hara & Co., Ltd.) or manually by an analyst blinded to time-of-day information (see Data analysis).

Open Field. Mice were placed into one of the corners of a 45 x 45 cm open field (O'Hara & Co., Ltd.), and their behavior was monitored for 5 min with an automated video tracking system. The time spent in the center of the open field (30% area, circular) and the distance traveled were determined using ImageJ software (NIH, MD, USA) with OpenField plug-in (O'Hara & Co., Ltd.).

Tissue preparation. For mRNA and protein analyses, mice were sacrificed by rapid cervical dislocation. Brain sections of 1-mm thickness containing the amygdala were prepared using a brain matrix (ASI Instruments, Warren, MI, USA), and basolateral and centromedial amygdalar complexes (BLA and CeA, respectively) were dissected using surgical knives, rapidly frozen, and stored at -80°C. For profiling SCOP expression in brain tissues, the BLA and CeA were dissected as described above, posterior hippocampi were cut out from the 1-mm-thick slice from which the amygdala nuclei were sampled, olfactory bulbs and

striatum were dissected[8], and a region containing the midbrain, cerebellum, pons, and medulla are combined ("posterior brain"). Bilateral tissue from each mouse were combined and treated as one sample (see below).

For the validation of *Scop* BLA KO, *Scop*$^{fl/fl}$ mice transduced with AAV-Cre or AAV-GFP were sacrificed, and brain sections were prepared as described above. The slices were then mounted on a glass slide and analyzed for GFP signal under a fluorescent stereoscopic microscope (Leica, Wetzlar, Germany). GFP-positive regions of the BLA were cut out using surgical knives while mounted on the microscope, rapidly frozen, and stored at -80°C until subsequent RNA extraction.

For fluorescent microscopy, AAV-injected mice were sacrificed by rapid cervical dislocation, and the brain was removed, mounted on a brain matrix (ASI Instruments), and sliced into 1-mm thick sections. The sections were analyzed for GFP signal under a fluorescent stereoscopic microscope (Leica).

Quantitative reverse transcription PCR. To quantitate mRNA levels, brain tissues were homogenized in TRIzol (Ambion, Oakland, CA, USA) using 27 gauge needles, and total RNA was extracted and purified using RNeasy minElute columns (Qiagen #74204, Valencia, CA, USA) according to the manufacturers' instructions. Extracted RNA was adjusted to 200 ng/µL, treated with 1U per µg RNA of DNaseI (Promega, Madison, WI, USA) for 30 min at 37°C, and reverse transcribed with GoScript Reverse Transcriptase (Promega) with an equimolar mixture of random hexamer and oligo-dT primers. Using the cDNA as a template, transcripts for *Rps29, Bmal1, Dbp, Scop,* and *Nr1d1* (*Rev-erb*α) were amplified with gene-specific primers using the GoTaq Master Mix (Promega #A6001) and a real-time qPCR thermal cycler (StepOne Plus, Life Technologies, MA, USA). Primers used for *Rps29, Bmal1* and *Dbp* have been previously described[9]. The primers for *Scop* were: for Fig. 3, 5'-

CTCCC ACCAA ACCTT CTCAT-3’ and 5’-GCAGG GTTTC CAGTT TGTTT-3’, which amplify a cDNA region spanning exons 10-11, and for Figs. 4&5, 5’-TTGAA CATCT GCCTG CCAAC-3’ and 5’-GGGGG TTTGC CTTAG GAAGT T-3’, which amplify a genomic/cDNA region within floxed exon 4. The primers for *Nr1d1* were: 5’-CGTTC GCATC AATCG CAACC-3’ and 5’-GATGT GGAGT AGGTG AGGTC-3’.

SDS-PAGE and Western blotting. BLA and CeA tissues were homogenized by syringing through 27 gauge needles in TNE buffer with NP-40 [20 mM Tris-HCl pH 7.4, 150 mM NaCl, 2 mM EDTA, 1% NP-40] with 5 mM EGTA, 50 mM NaF, 1 mM Na_3VO_4, 1 mM PMSF, and protease inhibitor cocktail (Complete EDTA-free, Roche, Basel, Switzerland). The homogenates were kept on ice for 15 min and spun at >10,000 x *g* for 15 min. The supernatants were subjected to SDS-PAGE and immunoblotting as previously described[7]. Signal was visualized using a conventional enhanced chemiluminescence detection system (GE Healthcare, Waukesha, WI, USA) and quantified using ImageJ software (NIH) by densitometric analyses based on relative standards loaded on every polyacrylamide gel. Immunoblotting conditions were as follows: αCB rabbit polyclonal antibody against SCOP[10], 1/1,500 in 3% BSA/TBST; mouse monoclonal antibody against β-actin (Sigma #A2228), 0.4 µg/mL in 3% skim milk (Difco, Detroit, MI, USA)/TBST; B1BH2 mouse monoclonal antibody against BMAL1[11], 1 µg/mL in 1% skim milk; blocking: 1 hr at RT, primary antibody: overnight at 4°C, secondary antibody: 2 hrs at RT.

Data analysis. Behavioral analyses were automated except for the elevated plus maze (EPM) test for wild-type C57BL/6J mice, for which all recorded behavioral data were shuffled into random orders, and entries into open and closed arms and the time spent on each arm were recorded by an operator blinded to time-of-day information for each subject. One-way ANOVA tests were used to examine the

statistical significance of data consisting of three or more groups. Unpaired two-tail Student's *t*-tests were used for analysis on data with two groups. All data are presented as means with SEM. *P* values are presented as “*P*” for ANOVA tests and “*p*” for *t*-tests.

References for Supplementary Information

1. Pisharath, H., Rhee, J. M., Swanson, M. A., Leach, S. D. & Parsons, M. J. Targeted ablation of beta cells in the embryonic zebrafish pancreas using E. coli nitroreductase. *Mech. Dev.* **124,** 218–229 (2007).
2. Shimba, S. *et al.* Deficient of a clock gene, brain and muscle Arnt-like protein-1 (BMAL1), induces dyslipidemia and ectopic fat formation. *PLoS ONE* **6,** e25231 (2011).
3. Iwasato, T. *et al.* Cortex-restricted disruption of NMDAR1 impairs neuronal patterns in the barrel cortex. *Nature* **406,** 726–731 (2000).
4. Kassai, H. *et al.* Rac1 in cortical projection neurons is selectively required for midline crossing of commissural axonal formation. *Eur J Neurosci* **28,** 257–267 (2008).
5. Berton, O. *et al.* Essential role of BDNF in the mesolimbic dopamine pathway in social defeat stress. *Science* **311,** 864–868 (2006).
6. Zhong, L. *et al.* Next generation of adeno-associated virus 2 vectors: point mutations in tyrosines lead to high-efficiency transduction at lower doses. *Proc Natl Acad Sci USA* **105,** 7827–7832 (2008).
7. Shimizu, K., Phan, T., Mansuy, I. M. & Storm, D. R. Proteolytic degradation of SCOP in the hippocampus contributes to activation of MAP kinase and memory. *Cell* **128,** 1219–1229 (2007).
8. Spijker, S. Dissection of rodent brain regions. *Neuroproteomics* **57,** 13–26 (2011).
9. Hirano, A. *et al.* FBXL21 regulates oscillation of the circadian clock through ubiquitination and stabilization of cryptochromes. *Cell* **152,** 1106–1118 (2013).
10. Shimizu, K., Okada, M., Takano, A. & Nagai, K. SCOP, a novel gene product expressed in a circadian manner in rat suprachiasmatic nucleus. *FEBS Lett* **458,** 363–369 (1999).
11. Yoshitane, H. *et al.* Roles of CLOCK phosphorylation in suppression of E-box-dependent transcription. *Mol Cell Biol* **29,** 3675–3686 (2009).

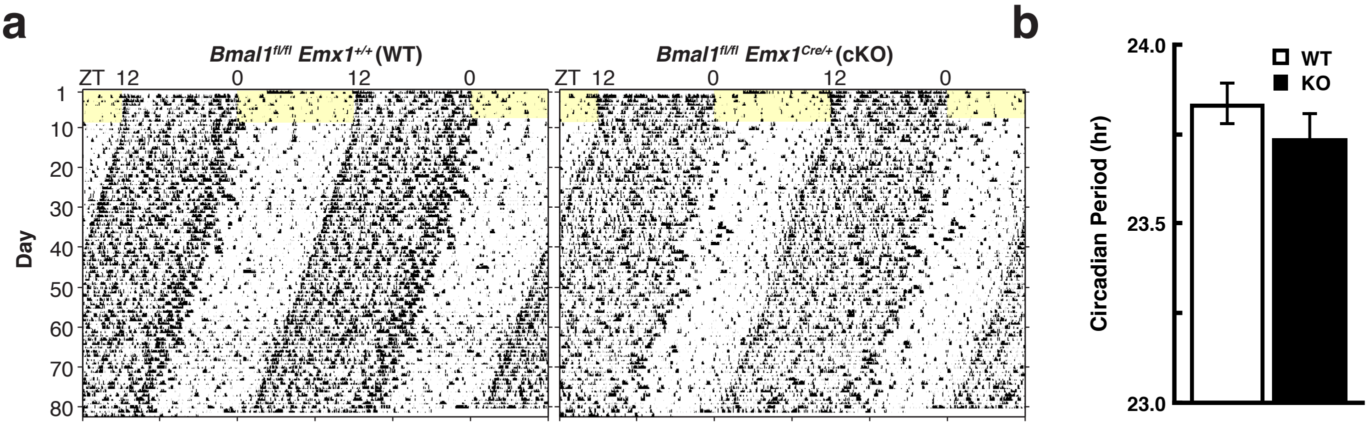

Supplementary Figure 1. Circadian activity rhythms of *Bmal1* conditional knockout (cKO) mice

a, Representative double-plotted actograms of *Bmal1* cKO and littermate control mice. Locomotor activity was recorded under an LD condition for 8 days then under a DD condition. Shaded areas denote the light period. ***b,*** Circadian periods of free-running activities under DD determined by the chi-square periodogram method. Error bars, SEM (n = 4 for WT, 5 for cKO).

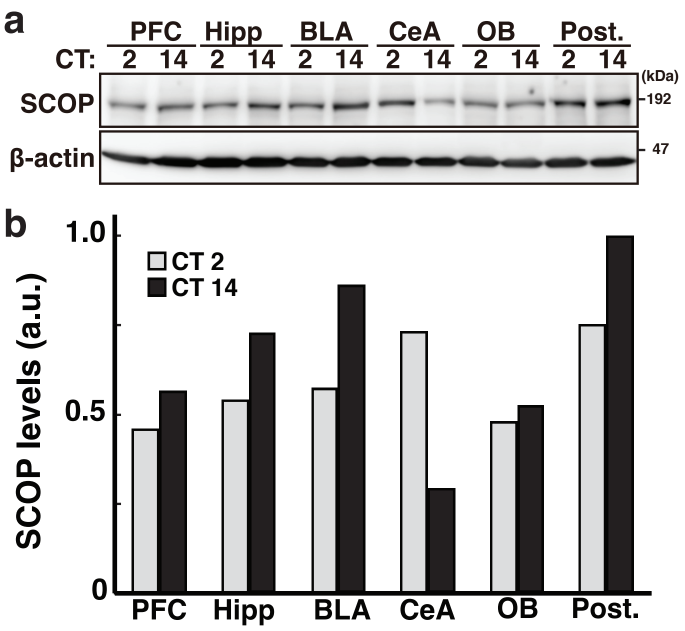

Supplementary Figure 2. SCOP expression in various brain regions

a,b, SCOP protein is widely expressed in the CNS (OB, olfactory bulb; PFC, prefrontal cortex; Hipp, hippocampus; CeA, centromedial amygdala; Post, posterior brain comprising midbrain, hindbrain, and cerebellum). ***a,*** Immunoblots against SCOP and β-actin using brain tissues sampled at CT2 and CT14. ***b,*** Quantification of (***a***) by densitometric analysis.

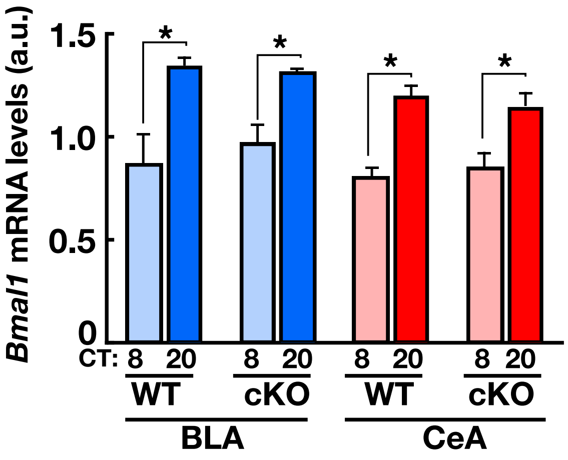

Supplementary Figure 3. Circadian expression of *Bmal1* mRNA in the amygdala of *Scop* cKO mice

The BLA (blue) and CeA (red) of *Scop* cKO mice and littermate WT mice sampled at CTs 8 and 20 were subjected to RT-qPCR analyses. Relative *Bmal1* mRNA levels are shown normalized to *Rps29*. $^*p < 0.05$ by unpaired Student's *t*-test. $n = 3$ per data point. Data are means with SEM.

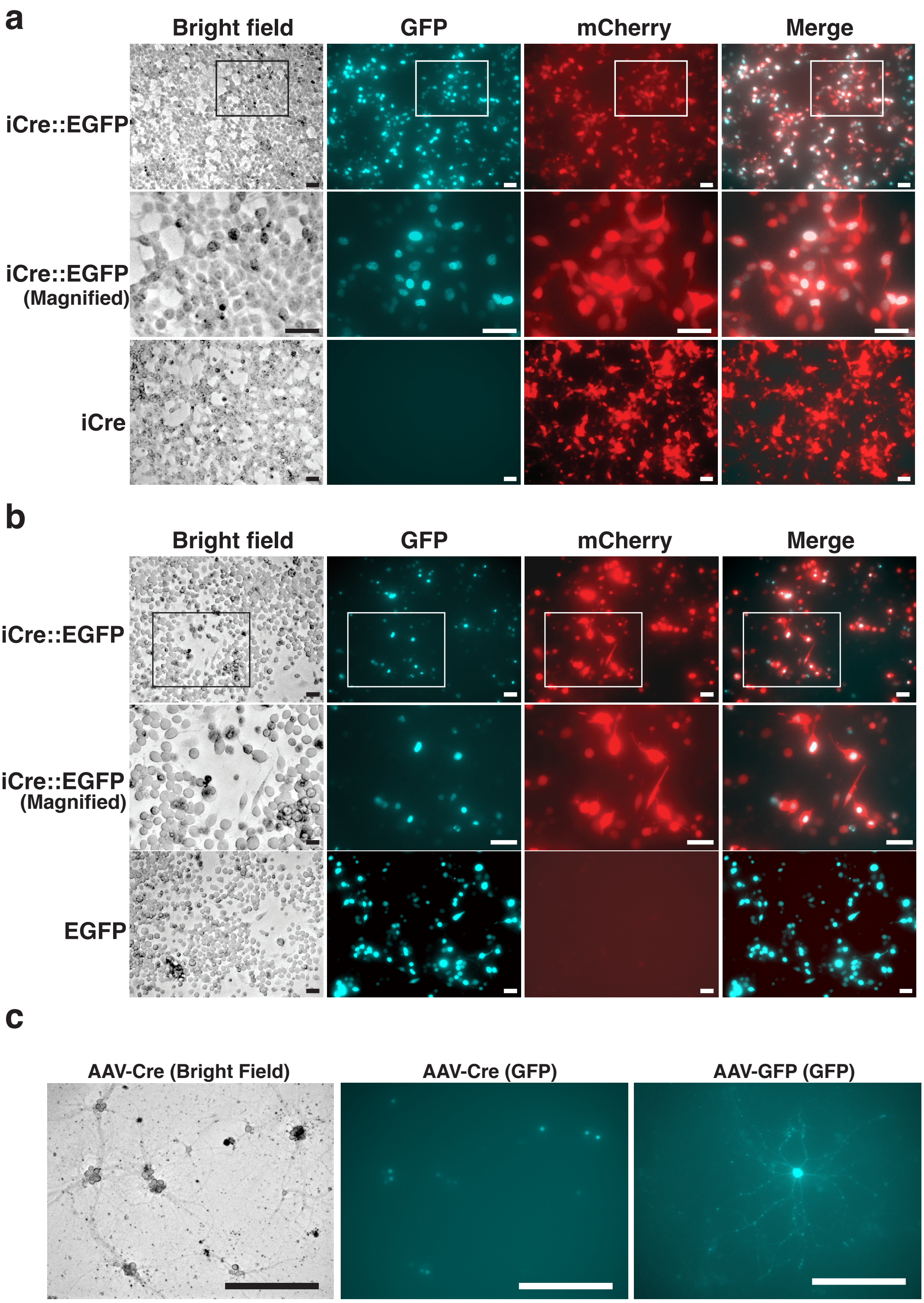
a
Bright field
GFP
mCherry
Merge
iCre::EGFP
iCre::EGFP
(Magnified)
iCre
b
Bright field
GFP
mCherry
Merge
iCre::EGFP
iCre::EGFP
(Magnified)
EGFP
c
AAV-Cre (Bright Field)
AAV-Cre (GFP)
AAV-GFP (GFP)

Supplementary Figure 4. Validation of AAV-Cre and AAV-GFP

a, Functionality of iCre::EGFP fusion construct was tested in HEK293T/17 cells. Transient expression of pCAG-iCre::EGFP, a plasmid construct expressing iCre::EGFP fusion protein under CAG enhancer/promoter, together with Cre reporter construct pCCALL2-mCherry (top and middle rows), which expresses mCherry in the presence of Cre recombinase, resulted in enhanced mCherry signals at levels comparable to cells transfected with pCAG-iCre (bottom row). Almost all GFP-positive cells were also mCherry-positive. ***b,*** Efficiency of human synapsin-1 (hSyn) promoter was tested in Neuro-2a cells. Transient expression of pAAV-hSyn-iCre::EGFP-WPRE, an AAV plasmid construct expressing iCre::EGFP under hSyn (Fig. S4*A*), together with pCCALL2-mCherry (top and middle rows) resulted in GFP and mCherry expression in transfected cells. mCherry-positive cells (representative of Cre recombinase activity) were not observed in cells transfected with pAAV-hSyn-EGFP-WPRE and pCCALL2-mCherry (bottom row). ***c,*** To test the infectious ability of the viral vectors in neuronal cells, primary hippocampal neurons were transduced with AAV-Cre (middle) or AAV-GFP (right), and GFP signal was examined 4 weeks after transduction. Cells with strong GFP signal were not observed in hippocampal neurons without the addition of viral vectors (not shown). A bright field image for cells transduced with AAV-Cre is also shown in the left. Scale bars: 50 μm. The fields of view magnified in middle rows in ***a,b,*** are shown as boxes in top rows.
